# A strong link between speed of visual discrimination and cognitive ageing

**DOI:** 10.1016/j.cub.2014.06.012

**Published:** 2014-08-04

**Authors:** Stuart J. Ritchie, Elliot M. Tucker-Drob, Ian J. Deary

**Affiliations:** 1 Centre for Cognitive Ageing and Cognitive Epidemiology, The University of Edinburgh, Edinburgh, UK; 2 Department of Psychology, The University of Edinburgh, Edinburgh, UK; 3 Department of Psychology, The University of Texas, Austin, TX, USA

## Abstract

Attempts to explain people’s differences in intelligence and cognitive ageing often hypothesize that they are founded substantially upon differences in speed of information processing [1]. To date, there are no studies that fulfill the design criteria necessary to test this idea, namely: having a large sample size; being sufficiently longitudinal; and using measures of processing efficiency that have a tractable biological basis, are grounded in theory, and are not themselves complex or based on motor response speed. We measured visual ‘inspection time’, a psychophysical indicator of the efficiency of the early stages of perceptual processing [2], in a large (*n* = 628 with full data), narrow-age sample at mean ages 70, 73, and 76 years. We included concurrent tests of intelligence. A latent growth curve model assessed the extent to which inspection time change is coupled with change in intelligence. Results showed a moderate correlation (*r* = 0.460) between inspection time performance and intelligence, and a strong correlation between change in inspection time and change in intelligence from 70 to 76 (*r* = 0.779). These results support the processing speed theory of cognitive ageing. They go beyond cross-sectional correlation to show that cognitive change is accompanied by changes in basic visual information processing as we age.

## Teaser

Theories of cognitive aging posit that old-age mental decline strongly relates to slower processing speed. Ritchie et al. examine data from older adults who had their IQ and fundamental visual discrimination speed measured as they aged. They find a robust link between decline in complex cognitive abilities and decline in basic perceptual speed.

## Main Text

The processing speed theory of cognitive ageing posits that a decline in the efficiency with which simple mental operations can be correctly completed is fundamental to ageing-related declines in higher cognitive functions [1]. Many studies have modeled the correlations of so-called processing speed measures with cognitive abilities such as spatial skill [3]. Typical studies use tests such as Digit-Symbol Substitution, a paper-and-pencil test, or reaction time, which measures decision response speed. Such measures cannot be assumed to be pure reflections of mental speed, as they are often contaminated with other processes such as memory [4], and rely upon physical reactions and movement speeds that may decline with age for non-cognitive reasons. Properly to test the processing speed hypothesis requires a speed assessment more fundamental than a paper-and-pencil or reaction time test.

**Figure 1:**
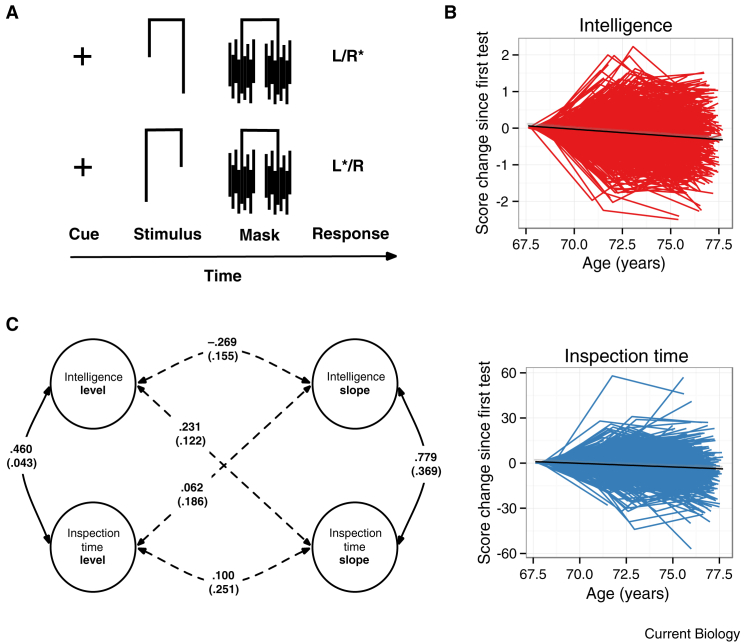
Stimulus description and model results. (A) The inspection time task. Participants focus on a cue, and are then shown one of the two possible stimuli, which is backward-masked after a brief exposure duration (see Supplemental Experimental Procedures). The participant then indicates whether the longer line was on the right or left side of the stimulus (L/R; correct responses marked with an asterisk). Responses are not timed; only their correctness is measured. (B) Individual trajectory plots with best-fit line (in black) showing each participant’s change from the initial test of intelligence and inspection time. (C) Path diagram of correlations between latent levels and slopes for intelligence and inspection time across the testing waves (see also Figure S1). Values are standardized path coefficients (SEs); dashed lines indicate non-significant paths.

To assess processing speed in this study, we used visual inspection time, a task based on psychophysical theory [5], with supporting functional brain anatomy [6], which has been linked cross-sectionally to intelligence [2]. This simple procedure (Figure 1A) requires the subject to discriminate between two lines of markedly different lengths. The stimuli are presented at several stimulus durations. At long durations, subjects make few errors, and as durations decrease, performance reduces to chance levels. The information available from the stimulus is a monotonically increasing function of its duration. Subjects with more efficient perceptual discrimination abilities are theorized to extract more information from brief displays, and thereby discriminate more successfully between the lines at shorter durations [2]. The task has been used in participants of all ages, including patients with dementia [7]. Responses are not timed; this reduces the task as far as possible to a pure test of perceptual discrimination efficiency. If the processing speed theory is correct, we would expect to find that change in inspection time correlates substantially with change in intelligence.

The inspection time task was administered to members of the Lothian Birth Cohort 1936 (initial *n* = 1,091) at three testing waves, when they were of approximate mean age 70, 73, and 76 years. At the same sessions, the participants completed four cognitive tests: Matrix Reasoning, Block Design, Letter-Number Sequencing, and Digit Span Backward, all of which measure ‘fluid’ aspects of intelligence that decline during old age [8]. A latent factor was extracted from the four tests to index general fluid intelligence (see Supplemental Experimental Procedures). At age 70, the correlation between inspection time and intelligence was *r*(1030) = 0.283, *p* < 0.001. At age 73, it was *r*(832) = 0.345, *p* < 0.001, and at age 76 it was *r*(642) = 0.369, *p* < 0.001 (Table S1). As illustrated in Figure 1B, there was significant decline with age in intelligence (−0.048 SDs/year, SE = 0.004, *p* < 0.001) and inspection time (−0.055 SDs/year, SE = 0.010, *p* < 0.001).

We modeled the data using a bivariate latent growth curve procedure (Supplemental Experimental Procedures, Table S2, and Figure S1), which produced the four latent variables shown in Figure 1C: these are the overall levels of inspection time performance and of intelligence across the three waves, and the slopes of their changes with age. Correlations between the four latent variables were calculated. The level of inspection time had a medium-sized correlation with the level of intelligence (*r* = 0.460, *p* < 0.001): as has been found in much prior work [2], those with higher intelligence had better inspection time performance (that is, more efficient speed of processing). More importantly, there was a large-sized correlation between the slope of intelligence change and the slope of inspection time change across the six years (*r* = 0.779, *p* = 0.035): those who declined more in intelligence declined more in visual processing speed (a standard deviation decline in intelligence was associated with 77.9% of a standard deviation decline in processing speed, and vice versa). These level–level correlations and slope–slope correlations were very similar in magnitude to those obtained when the same model was run with more conventional — more complex and less pure — measures of processing speed (Choice Reaction Time, Digit-Symbol Substitution, and Symbol Search) in place of inspection time, indicating that strongly coupled changes between processing speed and intelligence in old age are not simply artifacts of the memory, reasoning, or motor demands of the conventional measures. All the results survived several robustness checks (see Supplemental Results and Discussion).

Processing speed changes are strongly related to changes in higher mental abilities with ageing. This is true even when processing speed is measured by inspection time, a basic indicator of the efficiency of perceptual discrimination that does not depend on motor response speed, memory, or reasoning. The original theories of inspection time and intelligence proposed that basic speed of perceptual discrimination constitutes a limiting factor on more complex cognitive abilities [2,5]. Our finding that changes in perceptual discrimination had an appreciably larger correlation with cognitive decline than baseline perceptual discrimination had with baseline intelligence may indicate that ageing-related declines in speed of perceptual discrimination are especially relevant to the ageing-related declines in complex cognitive abilities, although they do not show that the direction of causation is necessarily from speed to cognition (‘bottom-up’) or from cognition to speed (‘top-down’; see Supplemental Discussion). Nevertheless, inspection time may be used as a ‘biomarker’ of cognitive decline [9,10]. These strong results encourage further investigation of the relation of perceptual efficiency to higher cognition, particularly in the context of cognitive ageing.

## Supplemental Information

Document S1. Supplemental Experimental Procedures, Supplemental Results and Discussion, Two Tables and One figure
