# Supplementary material for: A strong link between speed of visual discrimination and cognitive ageing
Source: Curr Biol. 2014 Aug 4;24(15):R681–3. doi: 10.1016/j.cub.2014.06.012 (PMC4123160; doi:10.1016/j.cub.2014.06.012)

Supplemental Information:
A strong link between speed of visual discrimination and cognitive ageing

Stuart J. Ritchie, Elliot M. Tucker-Drob, and Ian J. Deary

Inventory:

1. Supplemental Methods
1.1 Sample
1.2 Measures
1.3 Statistical Analysis
2. Supplemental Results
2.1 Descriptive Statistics
2.2 Age-associated change in cognitive tests
2.3 Measurement invariance for intelligence
2.4 Growth curve model results
2.5 Robustness checks
2.6 Other speed measures
3. Supplemental Discussion
4. Supplemental References
5. Supplemental Tables S1 and S2
6. Supplemental Figure S1

1. Supplemental Methods

1.1 Sample

The Lothian Birth Cohort 1936 is a sample of older individuals, most of whom completed a test of mental ability during the Scottish Mental Survey of 1947 [S1], and who were contacted in 2004-2007 for follow-up testing. The initial testing wave included 1,091 individuals (543 female) with an average age of 69.5 years (SD = .8). The second wave, carried out in 2007-2010, included 866 individuals (418 female) aged 72.5 (SD = .7). The third, carried out in 2011-2014, included 697 (337 female) aged 76.3 (SD = .7). The sample's recruitment, testing and findings are described in detail elsewhere [S2, S3]. All participants gave written informed consent before joining the cohort, and the study was approved by the Multi-Centre Research Ethics Committee for Scotland (MREC/01/0/56) and the Lothian Research Ethics Committee (LREC/2003/2/29).

1.2 Measures

Inspection time. The inspection time stimulus (illustrated in Figure 1A in the main article), presented on a computer monitor with a 160Hz refresh rate, consisted of a pair of parallel vertical lines, connected at the top with a 2.5cm-long horizontal line. One of the vertical lines was noticeably shorter than the other: 2.5cm compared to 5cm. All lines were 1.6mm wide. After seeing a fixation cross for 500ms, there was a gap of 800ms before participants were shown the stimulus, at one of a number of durations described below. This was followed by a 500-ms visual pattern mask made

up of several side-by-side vertical lines that obstructed both of the original lines. After mask-offset, participants were asked to indicate, by pressing the relevant button on the computer keyboard, which of the lines was the longer: right, or left. They could respond at their leisure; they were informed there was no need for a quick response. The task was programmed so that no responses would be accepted until mask offset, which prevented impulsive responses. There were 40 practice trials, which gave feedback about the correctness of each response. There were 150 test trials, with no feedback. For the test trials, the stimulus display duration varied; there were ten trials at each of the following fifteen durations: 6, 12, 19, 25, 31, 37, 44, 50, 62, 75, 87, 100, 125, 150 and 200ms (all to the nearest millisecond), presented in a random order. The final variable, used in all the analyses, was the total number of correct responses. Since participants all show a pattern where their performance is near-chance at the shorter durations and near-ceiling on the longer durations, individuals with a higher total number of correct responses are those who reach asymptote (100% correct) more rapidly, and thus are theorized to have faster perceptual processing speed. The total number of correct responses correlates strongly ($r \approx .7$) with the duration at which each participant's performance reaches asymptote, and moderately, though still substantially, with the duration at which their performance reaches 80% correct ($r \approx .5$).

Intelligence. Intelligence was measured using four non-verbal subtests of the Wechsler Adult Intelligence Scale, 3rd Edition UK (WAIS-III^{UK}) [S4]. First, Matrix Reasoning involves the completion of an array of patterns that are organized according to rules, and have a piece missing. When the participant has deduced the rule, they choose a piece from among a variety of responses to complete the array.

Second, Block Design involves the participant being shown a series of patterns on cards, and arranging blocks with different colors (white, red, or diagonally white and red) on each of their faces so that together they replicate the pattern. They are given two minutes to complete each pattern (though speed of response is not a part of the final score). Third, Letter-Number Sequencing, primarily a test of working memory, involves the participant listening to the tester reading a list of mixed letters and numbers of increasing length, and repeating back the numbers in numerical order then the letters in alphabetical order. Fourth, Digit Span Backwards also tests working memory. The tester reads out increasingly long lists of numbers, and the participant attempts to repeat them backwards.

Other speed measures. As discussed in the main document, we compared the performance of inspection time change as a predictor of intelligence change with three other speed tests. First, Digit-Symbol Substitution from the WAIS-III^{UK}, is a pencil-and-paper task where participants are shown a list of digits and their corresponding symbols, and have to then fill as many missing symbols to a presented list of digits as they can in two minutes. Second, Symbol Search, also from the WAIS-III^{UK}, involves participants examining lines of symbols and indicating whether designated target symbols are included, as quickly as possible. Third, mean four-Choice Reaction Time was measured with a dedicated device, described in detail elsewhere [S2]. After eight practice trials, participants had forty test trials where they were to press one of four keys indicated by numbers appearing on an LCD screen as quickly as possible.

1.3 Statistical Analysis

To plot individual trajectories in intelligence as shown in Figure 1B, we extracted a single factor from the scores on each of the four WAIS subtests with maximum likelihood estimation using the *factanal* function for R [S5], and used Bartlett's method for factor score estimation. This factor explained 44.9% of the variance in WAIS subtest scores across the three waves.

Growth curve modeling was performed in Mplus version 7.11 [S6]. The model, like other structural equation models, combines factor analysis and regression, allowing simultaneous estimation of latent intelligence factors, their change across time, and their relationship with the inspection time measurements. Mplus scripts for the model are available on request from the corresponding author.

A full path diagram of the bivariate growth curve model is shown in Figure S1. In the diagram, squares represent measured (observed) variables and circles represent latent (unobserved) variables. The connections between them are either single-headed arrows, indicating a directional relationship, or double-headed arrows, indicating a covariance. Covariance paths from a variable to itself represent residual variances.

In order to fit the growth curve model, some of the paths are freely estimated according to the data but others are fixed to particular values. In order to set the metric of the factor, the factor loadings for the first indicator is fixed at a constant value of one and the mean of the baseline factor is fixed at a constant value of zero. As is standard in growth curve modeling [S7], the paths from the latent intercept (or 'level') variables to each of the measures at waves 1, 2, and 3 are fixed at one,

meaning that the latent intercept variable represents an individual's expected level of performance at baseline. Customarily, growth models would then specify a fixed value for each of the paths from the latent slope parameters – in this case, zero, three, and six, for the time in years between the testing waves. This specifies a linear slope of change with age. However, since there was some variance in age at each wave (the participants were not all precisely 70, 73, and 76 years old at the three waves respectively), we allowed a finer-grained estimation of change across time by using the "TYPE IS RANDOM" option in Mplus to add individually-varying times for each participant to the paths from the latent slope variable.

The paths in Figure S1 marked 'LL' and 'SS' denote level-level and slope-slope correlations between intelligence and inspection time, respectively. These are the main paths of interest for our hypothesis. The paths marked 'LS' are within- and between-variable correlations between levels and slopes, with the specific variables denoted in subscript.

2. Supplemental Results

2.1 Descriptive statistics

Table S1 shows the correlations between each of the measures, along with their means and standard deviations.

2.2 Age-associated change in cognitive tests

The means of the two latent slope factors in the growth curve model, indicated significant declines in both intelligence and inspection time with age. As noted in the main article, overall intelligence declined by .048 SDs per year, SE = .004, $z = -12.184$, $p < .001$, and inspection time declined by .055 SDs per year, SE = .010, $z = -5.755$, $p < .001$. We also tested the change in the individual intelligence subtests by estimating a model with a univariate growth curve for each. This model showed that Matrix Reasoning declined by .036 SDs per year, SE = .005, $z = -6.641$, $p < .001$; Block Design by .045 SDs per year, SE = .004, $z = -10.239$, $p < .001$; Letter-Number Sequencing by .045 SDs per year, SE = .006, $z = -7.268$, $p < .001$; and Digit Span Backwards by .013 SDs per year, SE = .006, $z = -2.384$, $p = .017$. Thus, there was significant age-associated decline in each of the tests used in the final model.

2.3 Measurement invariance for intelligence

Since we modeled longitudinal change in a latent variable (intelligence), it was important to ensure that the same construct was being measured across time [S8]. We tested this by comparing the fits of models in which factor loadings and test intercepts were freely estimated to those in which they were constrained to be invariant across time.

The measurement model had acceptable fit to the data with no invariance (χ^2 = 313.31, Root Mean Square Error of Approximation = .080, Comparative Fit Index = .953, Tucker-Lewis Index = .921), metric invariance (only loadings invariant across time; χ^2 = 320.87, RMSEA = .075, CFI = .953, TLI = .931) and with strong factorial invariance (loadings and intercepts invariant across time; χ^2 = 358.89, RMSEA =

.074, CFI = .947, TLI = .932). Metric invariance could be imposed without a significant loss of model fit compared to no invariance, according to a chi-squared test (p = .27). Although the model with strong factorial invariance fit significantly more poorly than the model with metric invariance (p < .001), strong invariance was nonetheless preferable for the following reasons. First, the chi-squared test is over-powered in moderately large samples, and thus trivial differences will be classified as significant. Second, the RMSEA value for strong invariance (.074) was somewhat better than that for metric invariance (.075). Third, inspection of the freely-estimated intercepts indicated that they were very similar across time: for Block Design, they were 33.769, 33.982, and 33.039 for waves 1, 2, and 3 respectively; for Letter-Number Sequencing, they were 10.907, 11.011, and 10.675; and for Digit Span Backwards, they were 7.734, 7.897, and 7.986. We thus used the strong invariance model due to its superior interpretative value. It should be noted, however, that in a model with only metric invariance, a near-identical pattern of results was obtained.

2.4 Growth curve model results

Parameter estimates for the growth curve model are provided in Table S2. Unstandardized parameters are reported for all portions of the model except for the correlations among the level and slope factors, which we provide in the standardized metric for ease of interpretation. Note that, because the random effects approach implemented for our growth curve analysis specifies parameters to be person-specific, a single covariance matrix for the total population is not implied by the model. Fit statistics that are based on the comparison of the ‘model-implied’ population covariance matrix to the ‘observed’ sample covariance matrix (e.g. RMSEA, CFI, and

TLI) were reported for the measurement models above, but were not available for the full growth curve model.

As reported and discussed in the main text, there were substantial and significant correlations both between the level of intelligence and the level of inspection time (r = .460, SE = .043, z = 10.623, p < .001), and between the slope of intelligence and the slope of inspection time (r = .779, SE = .369, z = 2.109, p = .035). There were no significant associations between level and slope, either within or between variables.

2.5 Robustness checks

We ran four additional models to check the robustness of our findings. First, we adjusted each of the variables for sex before including them in the growth curve model. This made very little difference to the final model parameters (e.g. growth curve level-level correlation after controlling for sex = .447, p < .001; slope-slope correlation = .785, p = .035).

Second, to test whether pathological cognitive ageing influenced the results, we excluded all individuals with a score at the final wave lower than 24 on the Mini Mental State Examination (MMSE) [S9], a commonly-used cutoff for possible dementia. There were 12 individuals with scores below the cutoff at the final wave, and their exclusion resulted in a similar level-level correlation (.437, p < .001), and a still-strong and significant, slope-slope correlation (.670, p = .032).

The nature of the inspection time task is such that durations appear at random; short and longer durations appear unpredictably. Therefore, one may use performance on the longest durations as a possible check for inattention. In a third check for robustness, therefore, we modeled data from only those participants who scored 8 out of 10 or better (i.e. >=80% correct) on the trials with the two longest durations (150 and 200ms; ten trials each). 40 individuals at wave 1 had their inspection time scores removed for this reason, in addition to 45 individuals at wave 2, and 42 individuals at wave 3. Their exclusion hardly changed our results (level-level correlation = .424, $p < .001$; slope-slope correlation = .754, $p = .015$).

Our fourth and final robustness check was to change the way inspection time was characterized. Instead of using the "total number of correct responses" variable, we re-ran the analysis using a stimulus threshold value for each participant. To obtain this value, we calculated, for each individual, the shortest of the 15 possible stimulus durations at which they scored 80% correct or above (that is, at which they responded correctly 8 or more times out of 10). As noted above, the resulting variable correlates substantially with the "total correct" variable, but is not identical to it. Using this variable produced a numerically larger level-level correlation ($r = -.579$, $p < .001$; values are negative since shorter durations imply better inspection time performance) and slope-slope correlation ($r = -.854$, $p = .001$) than using the "total correct" variable. Thus, regardless of how inspection time was measured, we still obtained a substantial and significant correlation between the slopes of intelligence and inspection time.

2.6 Other speed variables

We tested the correlation between the slope of fluid intelligence and the slope of the three additional speed variables. For Digit-Symbol Substitution, the slope-slope correlation with intelligence was $r = .811$, SE = .228, $p < .001$. For Symbol Search, it was $r = .862$, SE = .288, $p = .003$. For Choice Reaction Time, it was $r = -.867$, SE = .186, $p < .001$ (note that lower scores on Choice Reaction Time indicate faster processing). Thus, the magnitude of the slope-slope correlation of inspection time with intelligence was similar to that of the other speed variables, even though inspection time is a far simpler measure.

3. Supplemental Discussion

Our results provide strong evidence for coupled change in speed of basic visual discrimination and fluid intelligence in later life. In this section, we discuss some points arising from the results that could not be included in the main paper for space reasons.

First, it should be noted that no significant correlations were found between the latent level parameters and the latent slope parameters, either within or between intelligence and inspection time. However, the level-slope correlation within intelligence was negative and near significance ($z = -1.734$, $p = .083$), indicating that individuals with higher intelligence scores tended to decline in intelligence more across time than those with lower scores. This may reflect the 'law of initial value' [S10], whereby there are more ways in which a high score can be reduced than there are ways in which a low score can be reduced (individuals with higher initial intelligence scores have "more to lose" than those with low initial intelligence). It may also reflect

regression to the mean [S11], though this is unlikely given our latent variable approach. Overall, then, even if this result were significant, it would be easily explainable in terms of well-known statistical regularities.

Second, we ran three additional models where the inspection time measure was replaced with, Digit-Symbol Substitution, WAIS Symbol Search, and Choice Reaction Time. The correlations of the slope of these tasks with the slope of intelligence were all around the same magnitude as the slope-slope correlation of inspection time and intelligence. These results strengthen our main finding, since they show that inspection time, an extremely basic task of visual discrimination ability that (as discussed in the main paper) is unlikely to be confounded by response speed, movement time, or more complex cognitive functions such as memory, is as strongly related to decline in higher functions as tests that are likely to suffer from these confounds. Expressed another way, these results demonstrate that longitudinal slope-slope correlations between processing speed and intelligence remain strong even when potential confounds to this relationship are removed.

Third, it is instructive to compare our results to previous attempts to elucidate the longitudinal relation of intelligence to inspection time. The most recent study of this relation was that of Gregory and colleagues [9], who showed in a sample of 124 participants over 70 that lower inspection time performance was predictive of more cognitive decline across an 18-month follow-up period, but that there was no association between longitudinal changes in inspection time and cognitive decline. The most likely reason for the difference between this previous result and our significant finding of a slope-slope correlation is the much longer follow-up period

for our analysis, though our larger sample (almost nine times larger at wave 1) and more complex modeling technique may also have allowed a more accurate estimation of the slope-slope correlation.

Finally, the processing speed theory of cognitive ageing predicts that a decline in speed underlies the decline in cognitive ability seen in later life [1]. Although our results show coupled change in the two parameters, our model is unable to test whether the direction of causality is from speed to intelligence (a ‘bottom-up’ process). An alternative theory, for instance, might state that declines in intelligence lead to poorer performance on the speed task (a ‘top-down’ process), that a complex transactional process takes place whereby the variables affect one another across time, or that a third (unmeasured) variable is the cause of decline in both factors. Although there are models that test at a finer-grained level the association of baseline performance on one task with later differences in another [S7, S12], they still do not provide a decisive test of the direction of causality, and this should be borne in mind when considering the theoretical implications of these (and similar) results.

4. Supplemental References

S1. Scottish Council for Research in Education. (1958). Educational and Other Aspects of the 1947 Scottish Mental Survey. London, UK: University of London Press.

S2. Deary, I.J., Gow, A.J., Taylor, M.D., Corley, J., Brett, C., Wilson, V., Campbell, H., Whalley, L.J., Visscher, P.M., Porteous, D.J. and Starr, J.M. (2007). The Lothian Birth Cohort 1936: A study to examine influences on cognitive ageing from age 11 to age 70 and beyond. BMC Geriatr. *7*, 28.

S3. Deary, I.J., Gow, A.J., Pattie, A., and Starr, J.M. (2012). Cohort profile: The Lothian Birth Cohorts of 1921 and 1936. Int. J. Epidemiol. *41*, 1576-1584.

S4. Wechsler, D. (1998). *WAIS-III-UK Administration and Scoring Manual*. London, UK: Psychological Corporation.

S5. R Core Team (2013). R: A language and environment for statistical computing. R Foundation for Statistical Computing, Vienna, Austria. Retrieved from: http://www.R-project.org/

S6. Muthén, L.K. and Muthén, B.O. (1998-2013). Mplus user's guide: The comprehensive modeling program for applied researchers. Los Angeles, CA: Muthén & Muthén.

S7. McArdle, J.J. (2009). Latent variable modeling of differences and changes with longitudinal data. Ann. Rev. Psychol. *60*, 577-605.

S8. Widaman, K.F., Ferrer, E., and Conger, R.D. (2010). Factorial invariance within longitudinal structural equation models: Measuring the same construct across time. Child Dev. Perspect. *4*, 10-18.

S9. Folstein, M.F., Folstein, S.E., and McHugh, P.R. (1975). "Mini-Mental State": A practical method for grading the cognitive state of patients for the clinician. J. Psychiatr. Res. *12*, 189-198.

S10. Wilder, J. (1957). The law of initial value in neurology and psychiatry: Facts and problems. J. Nerv. Ment. Dis. *125*, 73-86.

S11. Campbell, D.T., and Kenny, D.A. (1999). *A Primer on Regression Artifacts*. New York, NY: Guilford Press.

S12. Ghisletta, P., and Lindenberger, U. (2005). Exploring structural dynamics within and between sensory and intellectual functioning in old and very old age: Longitudinal evidence from the Berlin Aging Study. *Intelligence 33*, 555-587.

4. Supplemental Tables

Table S1. Correlation matrix with means and standard deviations for each variable measured at each testing wave. All variables correlated significantly at $p < .001$.

Variable	MR age 70	BD age 70	LNS age 70	DSB age 70	IQ age 70	MR age 73	BD age 73	LNS age 73	DSB age 73	IQ age 73	MR age 76	BD age 76	LNS age 76	DSB age 76	IQ age 76	IT age 70	IT age 73	IT age 76
MR age 70	-																	
BD age 70	.57	-																
LNS age 70	.44	.40	-															
DSB age 70	.40	.34	.54	-														
IQ age 70	.90	.84	.63	.48	-													
MR age 73	.65	.55	.35	.34	.67	-												
BD age 73	.52	.76	.38	.32	.70	.53	-											
LNS age 73	.37	.36	.62	.51	.49	.38	.36	-										
DSB age 73	.34	.31	.47	.64	.42	.35	.29	.55	-									
IQ age 73	.68	.73	.50	.45	.80	.89	.82	.59	.44	-								
MR age 76	.63	.56	.37	.37	.67	.64	.51	.36	.34	.67	-							
BD age 76	.55	.76	.40	.34	.72	.54	.76	.34	.29	.72	.57	-						
LNS age 76	.35	.29	.54	.50	.44	.30	.28	.66	.47	.44	.36	.32	-					
DSB age 76	.39	.30	.49	.65	.45	.35	.28	.52	.68	.43	.38	.32	.56	-				
IQ age 76	.67	.72	.49	.46	.79	.66	.68	.49	.43	.79	.90	.83	.56	.46	-			
IT age 70	.21	.27	.22	.17	.28	.17	.23	.23	.15	.25	.15	.27	.15	.15	.23	-		
IT age 73	.27	.29	.24	.17	.32	.26	.30	.30	.20	.35	.25	.33	.22	.18	.33	.59	-	
IT age 76	.27	.25	.21	.15	.31	.26	.26	.23	.14	.32	.29	.35	.25	.18	.37	.51	.59	-
N	1086	1085	1079	1090	1075	863	864	863	866	859	689	691	687	695	678	1041	838	654
Mean	13.49	33.79	10.92	7.73	.06	13.17	33.64	10.91	7.81	−.01	13.04	32.18	10.48	7.77	−.10	112.14	111.22	110.17
(SD)	(5.13)	(10.32)	(3.16)	(2.26)	(1.20)	(4.96)	(10.08)	(3.08)	(2.29)	(1.14)	(4.91)	(9.95)	(2.99)	(2.37)	(1.14)	(11.00)	(11.79)	(12.53)

Note: MR = Matrix Reasoning; BD = Block Design; LNS = Letter-Number Sequencing; DSB = Digit Span Backwards; IQ = intelligence; IT = inspection time

Table S2. Parameter estimates for each of the paths in the growth curve model shown in Figure S1. Note that all coefficients are unstandardized except those between the latent intercept and slope variables.

Path type	Path	*Estimate*	SE	*z*	*p*
Latent intercepts and slopes (standardized)	IQ intercept with IT intercept	.460	.043	10.623	< .001
	IQ intercept with IQ slope	−.269	.155	−1.734	.083
	IQ intercept with IT slope	.231	.122	1.892	.058
	IT intercept with IT slope	.100	.251	.397	.691
	IT intercept with IQ slope	.062	.186	.334	.738
	IQ slope with IT slope	.779	.369	2.109	.035
IQ factor loadings	MR	3.549	.141	25.179	< .001
	BD	7.149	.333	21.444	< .001
	LNS	1.989	.113	17.560	< .001
	DSB	1.287	.089	14.501	< .001
IQ subtest intercepts	MR	13.498	.143	94.203	< .001
	BD	33.724	.307	109.886	< .001
	LNS	10.901	.087	124.993	< .001
	DSB	7.836	.065	120.329	< .001
Residual variances	MR wave 1	12.532	.859	14.598	< .001
	BD wave 1	53.925	3.754	14.366	< .001
	LNS wave 1	5.810	.433	13.420	< .001
	DSB wave 1	3.301	.209	15.762	< .001
	MR wave 2	13.063	.944	13.842	< .001
	BD wave 2	55.675	4.041	12.605	< .001
	LNS wave 2	5.759	.457	12.605	< .001
	DSB wave 2	3.550	.235	15.114	< .001
	MR wave 3	12.324	.968	12.731	< .001
	BD wave 3	49.243	.254	14.536	< .001
	LNS wave 3	5.871	.466	12.586	< .001
	DSB wave 3	3.698	.254	14.536	< .001
	IQ factor	.042	.010	4.254	< .001
	IT	52.080	7.618	6.837	< .001
Residual covariances	MR wave 1 with MR wave 2	4.526	.805	5.619	< .001
	MR wave 1 with MR wave 3	3.970	.813	4.884	< .001
	MR wave 2 with MR wave 3	4.534	.830	5.461	< .001
	BD wave 1 with BD wave 2	31.971	3.327	9.609	< .001
	BD wave 1 with BD wave 3	29.428	3.448	8.536	< .001
	BD wave 2 with BD wave 3	30.019	3.391	8.853	< .001
	LNS wave 1 with LNS wave 2	2.380	.383	6.211	< .001
	LNS wave 1 with LNS wave 3	1.837	.370	4.961	< .001
	LNS wave 2 with LNS wave 3	2.946	.393	7.486	< .001
	DSB wave 1 with DSB wave 2	1.607	.189	8.48	< .001
	DSB wave 1 with DSB wave 3	1.640	.202	8.102	< .001
	DSB wave 2 with DSB wave 3	1.965	.211	9.304	< .001

Note: Factor loadings and intercepts for intelligence constrained to be invariant across time – see Section 2.3, above. Intelligence factors and inspection time scores had intercepts set to zero and invariant residual variances across all waves. Abbreviations: MR = Matrix Reasoning; BD = Block Design; LNS = Letter-Number Sequencing; DSB = Digit Span Backwards; IQ = intelligence; IT = inspection time

5. Supplemental Figure

Figure S1. Path diagram for the growth curve model. For full model description, see Section 1.3, Statistical Analysis, above. To allow individual variance in the time of testing, slope parameters are set to zero at the first testing wave (T0), time elapsed by the second wave (T1) and time elapsed by the third wave (T2). The model tests for a level-level correlation (path LL), a slope-slope correlation (path SS), and level-slope correlations within and between the IQ and IT variables (paths LS). Other abbreviations: IQ = intelligence; IT = inspection time; MR = Matrix Reasoning; BD = Block Design; LNS = Letter-Number Sequencing; DSB = Digit Span Backwards.

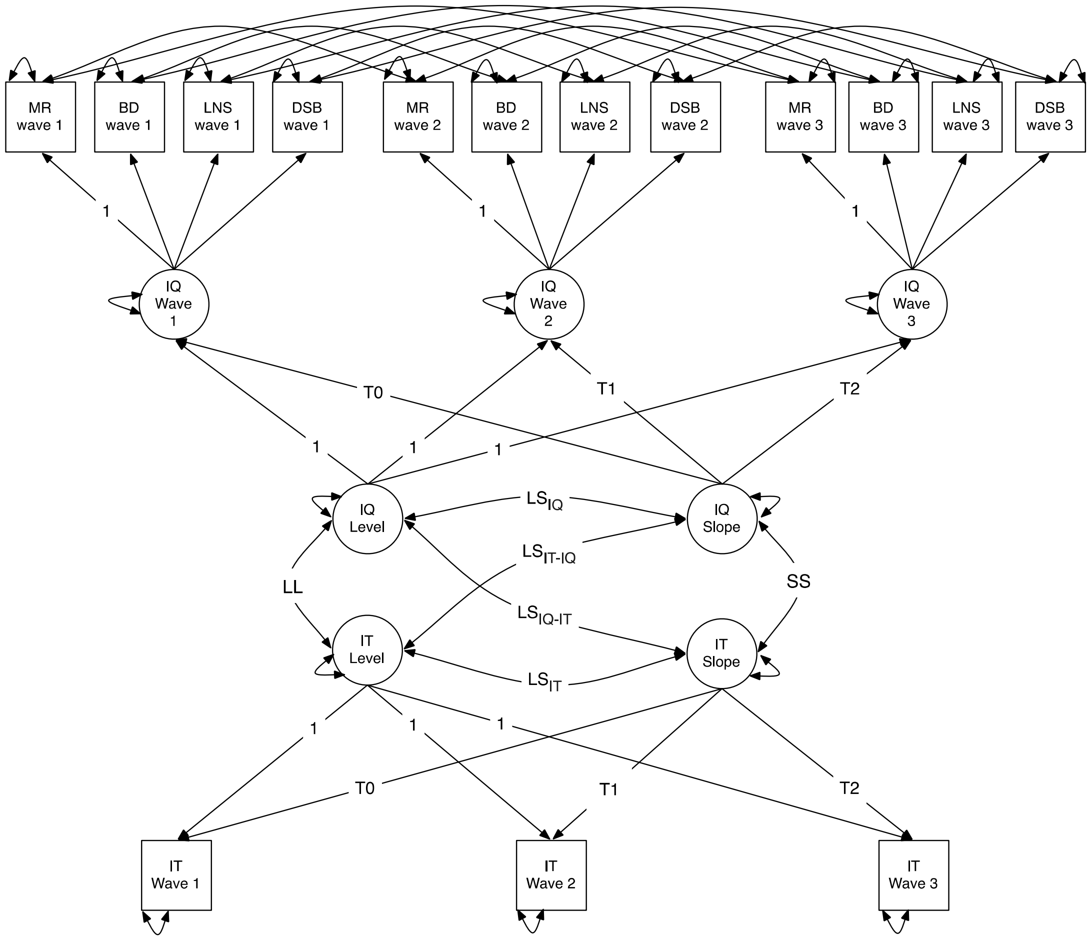

Supplement: Document S1. Supplemental Experimental Procedures, Supplemental Results and Discussion, Two Tables and One figure [file mmc1.pdf]
